# Axillary Cystic Lymphangioma in an Adult: A Case Report

**DOI:** 10.7759/cureus.55390

**Published:** 2024-03-02

**Authors:** Archana Khanduri, Deepak Gusain, Nalini Bansal, Jyoti Koli, Rahul Gupta

**Affiliations:** 1 Gastrointestinal Surgery, Synergy Institute of Medical Sciences, Dehradun, IND; 2 Pathology, Institute of Gastro and Hepatopathology (IGHP), Gurugram, IND; 3 Internal Medicine, Indresh Hospital, Dehradun, IND

**Keywords:** benign cyst, magnetic imaging resonance, open surgical excision, axillary cyst, cystic lymphangioma

## Abstract

Lymphangioma is a congenital malformation of the lymphatic system most often reported in children. Its occurrence in adults is rare. It usually develops in the head, neck, and axillary region. It mimics other conditions, such as cold abscess, simple cyst, hydatid cyst, and hemangioma, on clinical examination. Here, we report a case of cystic lymphangioma in the axillary region of a 32-year-old male. The patient underwent surgical excision and histopathology confirmed cystic lymphangioma. Although it is very rare in adults, cystic lymphangioma should be considered in the differential diagnosis of an axillary mass.

## Introduction

Cystic lymphangioma was first described by Redenbacker in 1828 [1]. It is a benign lymphatic malformation typically found in children [2]. It has been included in the revised International Society for the Study of Vascular Anomalies (ISSVA) 2018 classification [3]. The exact cause is not known but the proposed mechanisms include congenital weakness of the lymphatic walls, proliferation of the lymphatic vessels, and blockage of the lymphatic channels. In neonates and infants, the diagnosis is straightforward due to its typical location, appearance, and clinical findings. However, cystic lymphangiomas in adults are uncommon, with only case reports and series being published in the English literature [4-11]. Lymphangioma can occur anywhere in the body. The most commonly reported sites are the posterior triangle of the neck (75%), axilla (20%), mediastinum (5%), groin, retroperitoneal space, and pelvis [6]. Its occurrence in the axilla is rare with very few cases reported worldwide [5,6,8,10,11]. Here in, we report an unusual case of a large cystic lymphangioma in an adult male, which was treated successfully by surgical excision.

## Case presentation

**Figure 1:**
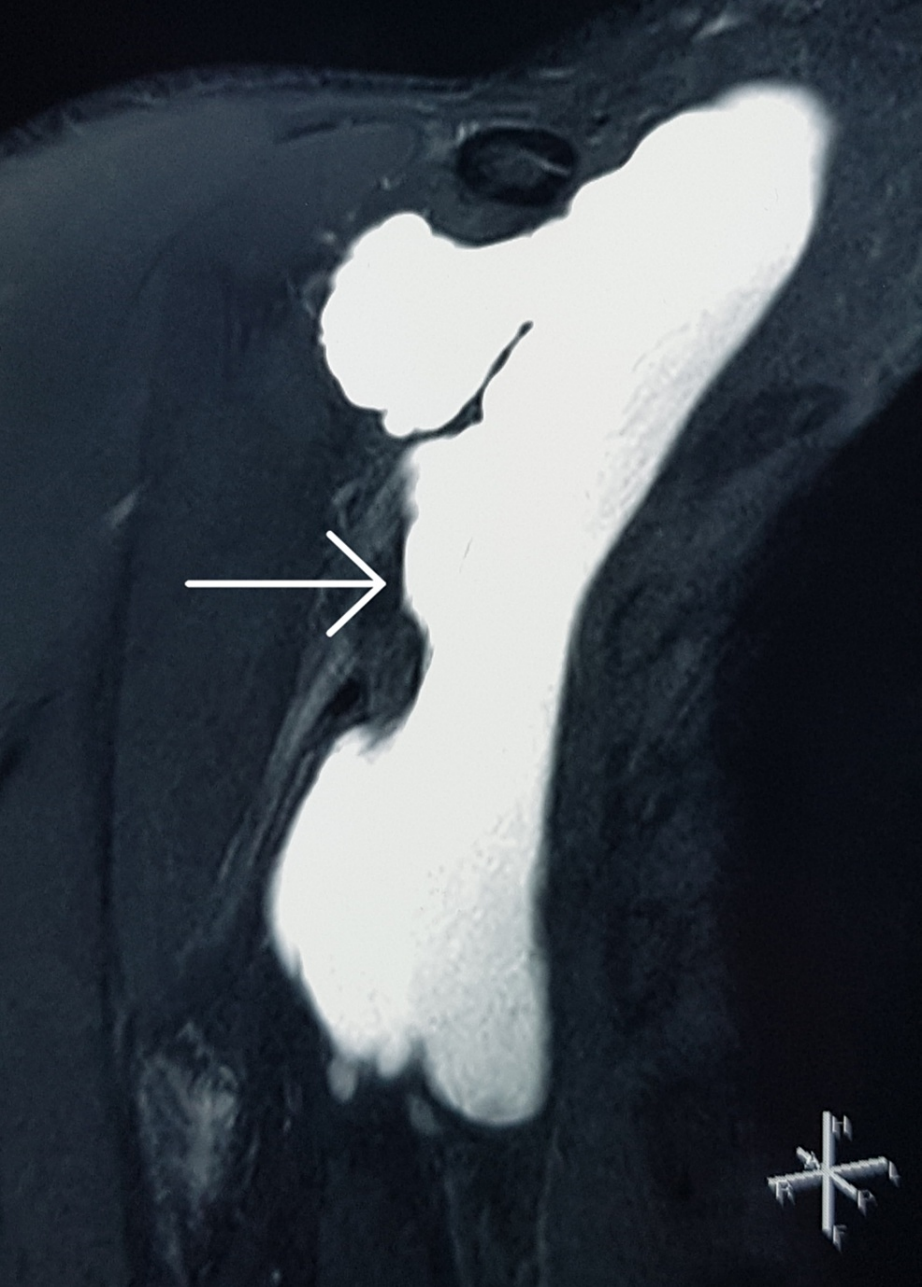
Magnetic resonance imaging (coronal section) showing the hyperintense lobulated cystic lesion in the right axilla on T2 sequence with no solid components.

A 32-year-old man with no comorbidities presented with swelling in the right axillary region for two months. The swelling was gradually increasing in size and was associated with mild pain. Ultrasound of the right axilla performed at another hospital revealed a large lobulated 9.5 x 6.7 x 5.0 cm cystic lesion with a volume of 170 cc. Subsequently, he underwent ultrasound-guided percutaneous aspiration at the same hospital, where 100 ml of clear fluid was aspirated. The details of the fluid analysis were not available. The swelling recurred within one week after the aspiration. Subsequently, he presented to our hospital for definitive treatment. On clinical examination, there was an 8 x 7 x 5 cm cystic swelling in the right axilla. The swelling was soft, non-pulsatile, non-tender, and separate from the overlying skin. Magnetic resonance imaging (MRI) of the right axilla revealed a large, lobulated, well-defined, thin-walled cystic lesion with no obvious solid component in the right axillary region with the same dimensions as described before, suggestive of lymphangioma (Figure 1). The lesion was in close relation with the axillary neurovascular bundle with preserved fat planes.

**Figure 2:**
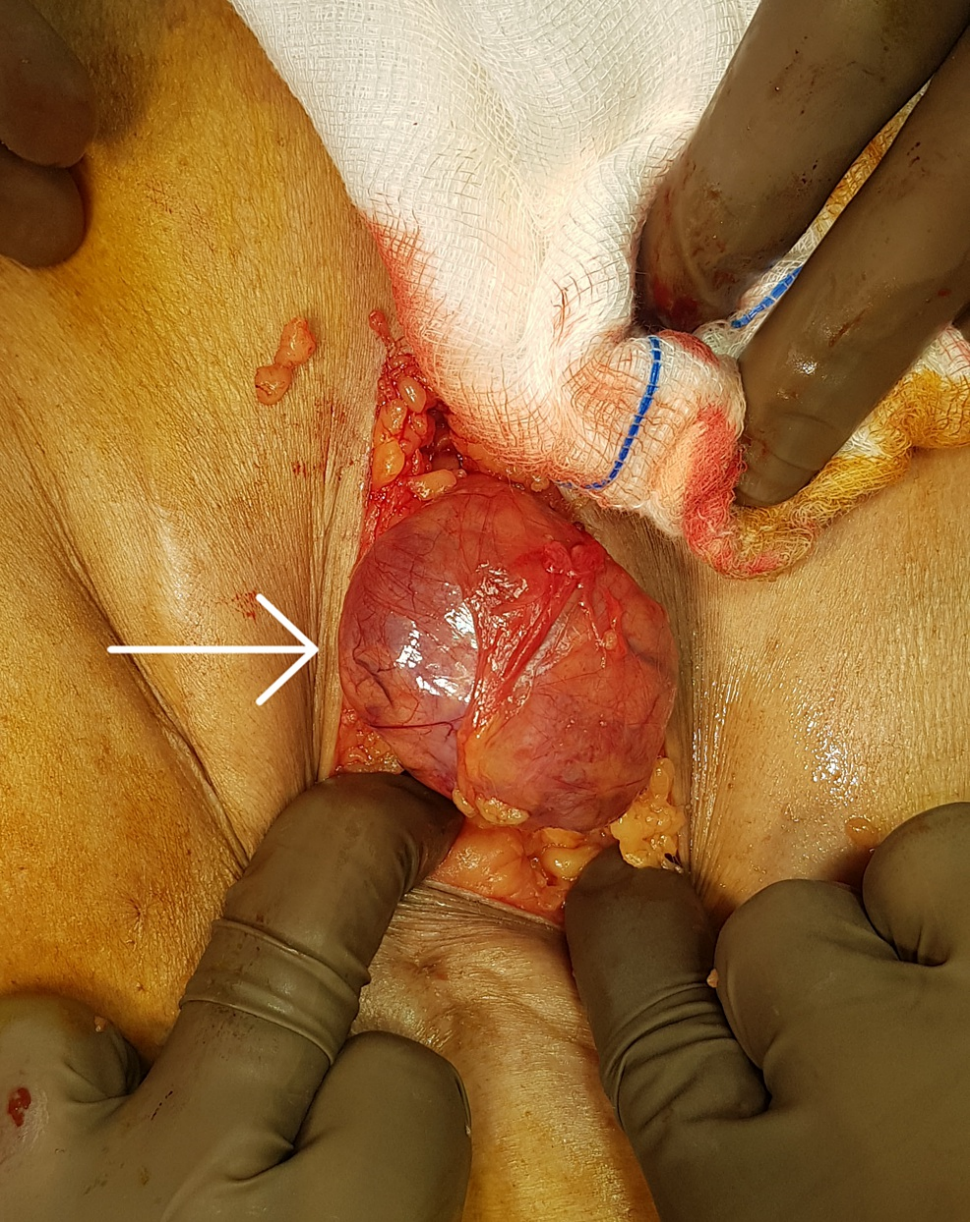
Intraoperative photograph showing the cystic lesion in the right axilla

Based on these findings, surgical excision of the right axillary cyst was performed under general anesthesia. Intraoperatively, a 10 x 8 x 6 cm cystic lesion in the right axilla containing serosanguinous fluid was present (Figure 2). The cystic lesion was completely excised up to the apex of the axilla. Multiple enlarged axillary lymph nodes were present, which were excised for histopathological examination. The wound was primarily closed over a suction drain. The operative time was 80 minutes with an estimated blood loss of 50 ml and a hospital stay of one day. The drain was removed on postoperative day 3.

**Figure 3:**
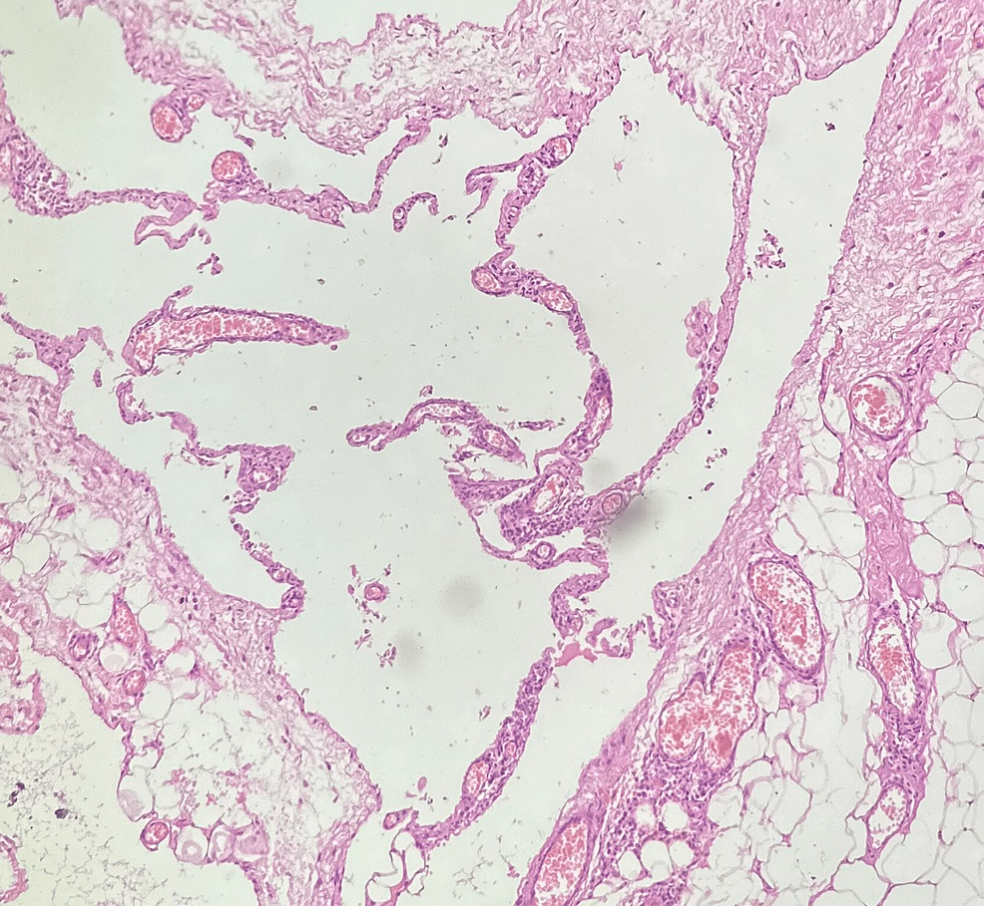
Histopathological examination of the cyst wall revealed a benign epithelial lining with surrounding adipose tissue (hematoxylin & eosin, 20x)

The smear examination of the cyst fluid revealed predominantly lymphocytes. The cyst fluid protein levels were 5.44 gm%, sugar levels of 106 mg%, and lactate dehydrogenase level of 300 IU/L. The cyst fluid adenosine deaminase level was 4 IU/L. The cyst fluid had no growth of aerobic culture and tuberculosis-polymerase chain reaction (TB-PCR) was negative. The histopathological examination of the cyst wall revealed dilated empty spaces lined by flattened epithelial cells with focal aggregates of lymphoid cells suggestive of benign cystic lesion morphologically compatible with lymphangioma with no evidence of malignancy (Figure 3). Axillary lymph node biopsy revealed reactive hyperplasia. There was no evidence of recurrence at 15 months after surgery.

## Discussion

Lymphangiomas are uncommon, slow-growing, benign lymphatic malformations [5,6]. Most of them are congenital in nature. Hence, they are most frequently reported in children less than two years of age [6,9]. However, the exact etiology of adult lymphangioma is not clear. The proposed pathophysiological mechanisms include blockage of the lymphatic channels with proliferation of the lymphatic vessels secondary to trauma, infection, pregnancy, or iatrogenic factors [6,8,12]. Recently, dysregulation of amphiregulin has been found to be associated with the development of cystic lymphangioma in the mice model [13].

The most widely used classification of lymphangioma divides it into microcystic (< 2 cm^3^), macrocystic (> 2 cm^3^), and mixed lesions based on the size of the cysts within the lymphangioma [5,9]. Some authors also classify them as capillary, cavernous, and cystic [5]. Capillary lymphangioma consists of small-sized capillaries and thin-walled vessels [8]. Cystic lymphangiomas are characterized by spaces filled with clear fluid and have well-defined cysts. Cavernous lymphangiomas, on the other hand, consist of lymphatic channels that are dilated and contain lymphoid aggregates [8,11].

Lymphangioma can be difficult to diagnose preoperatively, as it appears like other conditions, such as cold abscess, simple cyst, synovial cyst, hydatid cyst, cystic sarcoma, and hemangioma, on clinical examination [4,6].

Ultrasound is the initial modality of choice to look for the size, shape, location, depth, and contents of a soft tissue cystic mass. Cystic lymphangioma typically appears as a multiloculated hypoechoic mass with thin septae [5,8]. There may be hyperechoic contents within the cyst or its wall in case of infection, bleeding, or calcification. Computed tomography is useful for determining the anatomical location, size, and extent of the retroperitoneal and intra-abdominal cystic masses and their relation to the adjoining vessels and bowel loops [7]. MRI is the best imaging modality to study a cystic soft tissue mass in the head, neck, and extremities. It helps visualize thick enhancing septae seen in cystic sarcoma, daughter cysts, which are characteristic of hydatid cysts, and blood and solid debris within the cysts. Cystic lymphangioma appears as a hyperintense lesion on T2- T2-weighted images and hypointense on T1-weighted images with thin non-enhancing septae [9,11].

All three subtypes of lymphatic malformations are typically managed in a similar manner, with complete surgical removal resulting in the lowest rate of recurrence [7,8,9,11]. However, depending on the size, location and clinical presentation of the malformation, percutaneous aspiration with the injection of sclerosing agents (sclerotherapy) may also be considered as an alternative treatment [9,11]. The sclerosants reported in the literature are OK-432, bleomycin, doxycycline, acetic acid, alcohol, and hypertonic saline [11].

The main concern with the treatment of cystic lymphangioma is the risk of recurrence. Simple aspiration and sclerotherapy have been associated with a high recurrence rate but low complications [7,11]. On the other hand, surgical treatment of lymphangioma has the lowest recurrence rate but potential complications such as intraoperative adjoining neurovascular injury, seroma formation, etc. [7,11]. Indocyanine green fluorescence imaging system has been recently used to allow complete excision of the cyst with minimal damage to the surrounding structures especially for axillary cystic lymphangioma [8].

## Conclusions

The present case emphasizes the need for considering cystic lymphangiomas in the differential diagnosis of axillary lesions in adults. Despite the availability of advanced imaging techniques, the diagnosis of adult lymphangiomas still presents a challenge. The exact cause of their development in adults is not known but the literature suggests that trauma can trigger the development of cystic lymphangiomas in adults. Surgical excision is associated with the lowest recurrence and allows obtaining tissue for making a definitive diagnosis by histology.
